# Unraveling the Enigma: A Report on a Rare Case of Cutaneous Horn, an Extraordinary Dermatological Occurrence

**DOI:** 10.7759/cureus.41987

**Published:** 2023-07-17

**Authors:** Siddharth Sankar Das, Suhasini Krishnan, Susmita Das, Khalifa Almheiri, Tariq Abdul Hamid

**Affiliations:** 1 General Surgery, Dubai Hospital, Dubai, ARE; 2 Medicine, Dubai Academic Health Corporation, Dubai, ARE; 3 Obstetrics and Gynaecology, Aster DM Hospital, Dubai, ARE; 4 Dermatology, Dubai Academic Health Corporation, Dubai, ARE; 5 Urology, Dubai Hospital, Dubai, ARE

**Keywords:** projection, excision surgery, abdomen, horn, sebaceous horn, cutaneous horn

## Abstract

A cutaneous horn is a rare, hyperkeratotic, projecting lesion that can be mostly found in sun-exposed areas of the skin. The base of the lesions can reveal an underlying malignancy. They can also be associated with several benign or pre-malignant dermatologic conditions. A biopsy of the base of the lesion and histopathological analysis are needed to confirm the diagnosis. Management depends on the underlying disease; however, surgical excision is the preferred treatment method.

## Introduction

A cutaneous horn, less commonly described as cornu cutaneum or devil's horn, is a conical, protruding, hyperkeratotic growth, sometimes large enough to resemble an animal horn [1]. Compacted keratin forms the horn's main scaffolding, and several skin lesions can be seen at its base [2]. The origin of these lesions is the basal keratinocytes and can be associated with benign, pre-malignant, or malignant processes, particularly in sun-exposed areas of the skin [3]. On clinical examination, one can typically find a straight or curved, white or yellow-brown exophytic, circumscribed projection from the skin; however, there can be several variations in color, size, and shape [1]. Some conditions associated with this lesion are actinic keratosis, sebaceous molluscum, verruca, and malignancies such as squamous cell carcinoma, malignant melanoma, and basal cell carcinoma [4]. The prevalence and incidence of the condition have not been reported. Commonly seen in middle-aged males, in more overexposed areas like the nose, pinna, forehead, scalp, back of the arm, and forearm, but rarely, the anterior abdominal wall as found in this patient [5,6]. Due to their clinical ambiguity and potential malignancy, these lesions must undergo biopsy from the base for histopathological analysis. Only then can subsequent, appropriate treatment be initiated [2].

## Case presentation

**Figure 1:**
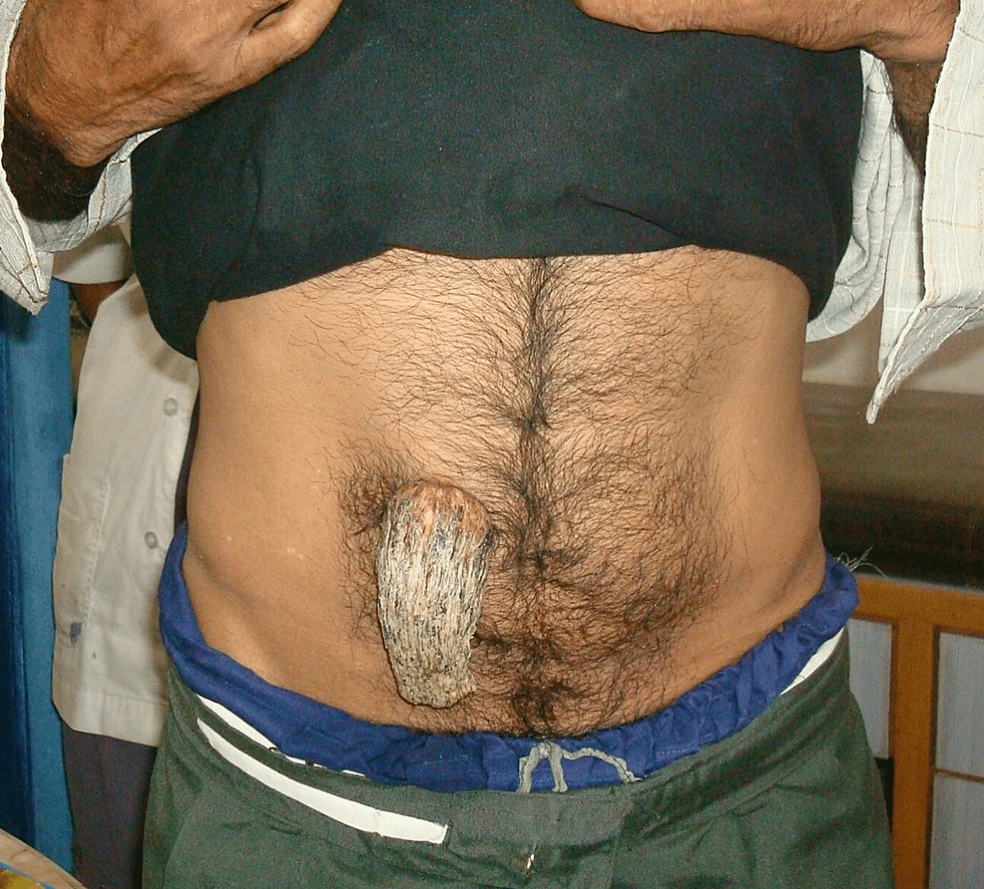
Cutaneous horn arising from the anterior abdomen (anterior view)

A 34-year-old South-Asian male, a small shop owner, visited the general outpatient surgery clinic with a strange history of a horn-like projection from his abdomen (Figure 1) for around 25 years. As per the patient, it started as a smooth, hard, three- to four-millimeter projection from the right side of his abdomen. It was hard but painless to touch, slowly growing to the present size of around 12 cm curved length. He denied any history of trauma to his abdomen since childhood and did not give any history of similar lesions in any other part of his body. He denied any significant medical or surgical history except for the unusual growth. He reported frequent outdoor activities as per his work. He has normal Indian dietary habits. He delayed his visit to any physician for checkups in his early life due to shyness. He was easily able to hide the growth under his clothes. His concern grew as he planned to get married, and he sought consultation for the growth.

**Figure 2:**
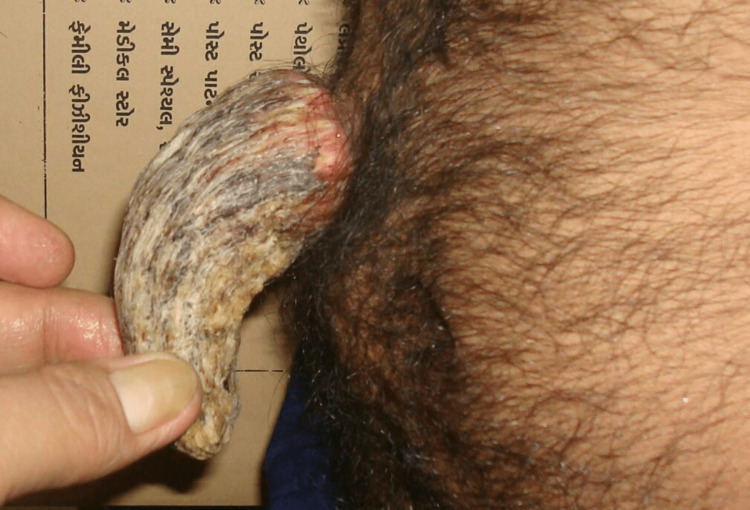
Lateral view of the cutaneous horn arising from the anterior abdomen

The patient had normal physical growth with a muscular body on examination. Upon closer inspection of the abdomen, a curved growth of around 12 cm x 5 cm x 4 cm in length, width, and height, respectively, was seen arising from the anterior abdomen, around 10 cm cranial to the level of the umbilicus and one-inch right lateral to the midline (Figure 2). 

The lesion was painless, firm-to-hard on palpation, and projected more outward as a curved horn on the leg-rising test's contraction of the abdominal wall muscles. No features of malignancy, like adjacent surrounding skin involvement or changes in skin texture, were noted. No similar growth was noticed on his body. Despite thoroughly discussing treatment options and possible outcomes, the patient hesitated for an excisional biopsy as he believed the growth to be auspicious.

## Discussion

Patients with cutaneous horns usually present with a conical, projecting growth in predominantly sun-exposed areas. Therefore, it is important to inquire about the patient's nature of work or involvement in outdoor activities [2,6]. It should be noted that these lesions can appear anywhere on the skin or mucosa, regardless of sun exposure [1]. Some patients may present with complaints of pain at the site of the lesion; however, this is not always the case [3,7]. A positive history for both factors should raise suspicion of an underlying malignant process [1].

Upon palpation, the clinician would find a firm-to-hard, well-rooted structure varying from a few millimeters to several centimeters long. The horns are usually prominent, white to yellowish, and keratinous [2,3]. The clinician could find a nodule, crateriform, or flat base at the base of the lesions. Malignancy should be suspected if signs of inflammation are present [1]. The pathophysiology and histopathological findings of the condition depend on the underlying disease [6]. If benign, the lesion slowly grows from months to years. However, in malignancy, rapid mitotic activity can accelerate the growth of the horn. It has also been reported that those lesions with a wider base are associated with malignant underlying disease processes [5,7].

Histopathological findings of the horn will show thickening of the stratum corneum or hyperkeratosis with or without acanthosis. If the lesion is benign, one can observe parallel, horizontal layers of keratinized tissue [8]. While a clinical diagnosis can be sufficient, the standard of care involves a biopsy of all cutaneous horns for confirmation. Those lesions with suspected malignancy should undergo a biopsy, and the need for a biopsy must be explained to every patient if they show reluctance toward surgical excision. As the conical part of the cutaneous horn mostly consists of compacted keratin, the base of the lesion is the area that would reveal the mitotic activity and confirm malignancy or pre-malignancy condition [3,5].

Management of a cutaneous horn varies as per the underlying etiology of the condition. There are three mainstays of treatment: surgical, through total excision, medical therapy, or laser ablation [1]. In the case of benign lesions, observation can be employed as the initial management, or the lesion may be surgically removed for cosmetic reasons upon the patient's request. Regular follow-up for evaluation of cutaneous horn growth is advised, and wide local excision is recommended for pre-malignant or confirmed malignant cases [4]. Determination of the margins for these cases should be made following the latest treatment guidelines [9]. Ablative lasers or electrocautery are other options used for small lesions and mainly for cosmetic purposes with patient's demand. It is not considered an alternate method for excisional biopsy [3]. Patients with confirmed squamous cell carcinoma or basal cell carcinoma should undergo evaluation for metastasis, particularly assessment of regional lymphadenopathy and lymph node biopsy [1,3].

Psychological aspects should also be considered in the case of a cutaneous horn. Some patients may express feelings of embarrassment and believe the lesion to be a hindrance to their social life. The clinician must address these aesthetic complaints and counsel the patient as they can significantly impact their mental health [10-12].

## Conclusions

The cutaneous horn of the abdomen is a rare and peculiar occurrence. However, clinical history, examination, and excisional biopsy confirm the diagnosis. Successfully managing a cutaneous horn involves a combination of surgical excision and close follow-up. Regular monitoring is crucial to identify any potential recurrence or malignant transformation. More reporting of such cases and research will contribute to a better understanding of this fascinating phenomenon and aid in optimizing patient care.
